# Chromosomal Microarray Analysis and Karyotype Analysis for Prenatal Diagnosis of Fetuses With Abnormal Ultrasound Soft Markers

**DOI:** 10.1002/jcla.70033

**Published:** 2025-04-07

**Authors:** Lina Liu, Lingna She, Zhiyuan Zheng, Shuxian Huang, Heming Wu

**Affiliations:** ^1^ Department of Ultrasound Meizhou People's Hospital, Meizhou Academy of Medical Sciences Meizhou China; ^2^ Department of Prenatal Diagnostic Center Meizhou People's Hospital, Meizhou Academy of Medical Sciences Meizhou China; ^3^ Meizhou Municipal Engineering and Technology Research Center for Molecular Diagnostics of Major Genetic Disorders Meizhou People's Hospital, Meizhou Academy of Medical Sciences Meizhou China

**Keywords:** chromosomal microarray analysis, copy number variation, karyotype, prenatal diagnosis, ultrasound soft markers

## Abstract

**Objective:**

To explore and evaluate the value of chromosomal microarray analysis (CMA) in fetuses with abnormal ultrasound soft markers.

**Methods:**

A retrospective study was conducted on 193 fetuses with abnormal ultrasound soft markers who received prenatal diagnosis at Meizhou People's Hospital, between October 2022 and February 2024. Genetic detection of fetal specimens obtained by ultrasound‐guided puncture was carried out. The detection rates of karyotype analysis and CMA for chromosomal abnormalities in different ultrasonic abnormalities were analyzed.

**Results:**

Of the 193 fetuses, there were 77 (39.9%) fetuses with increased nuchal translucency(NT) thickness, 33 (17.1%) with ventriculomegaly, 29 (15.0%) with nasal bone hypoplasia, followed by choroid plexus cyst, pyelic separation, echogenic bowel, single umbilical artery, with persistent left superior vena cava, and persistent right umbilical vein. Aneuploidy was mainly found in fetuses with increased NT thickness or and nasal bone hypoplasia, while P/LP CNVs were mainly concentrated in fetuses with increased NT thickness or ventriculomegaly. The detection rate of karyotype was 5.7% (11/193), the detection rate of aneuploidy plus P/LP CNVs in fetuses with abnormal ultrasonic soft markers by CMA was 10.9% (21/193), and the additional detection rate of CMA was 5.2%.

**Conclusions:**

CMA can significantly improve the detection rate of chromosomal abnormalities in fetuses with abnormal ultrasonic soft markers compared with karyotype analysis. There was a significant difference in detection rates of chromosomal abnormality between CMA and karyotype analysis in the single ultrasonic abnormality group, but none in the multiple ultrasonic abnormalities group.

## Graphical abstract

Genetic detection of fetal specimens obtained by ultrasound‐guided puncture was carried out using karyotype analysis and chromosomal microarray analysis (CMA) in this study, and the detection rates of chromosomal abnormalities in different ultrasonic abnormalities were analyzed. CMA can significantly improve the detection rate of chromosomal abnormalities in fetuses with abnormal ultrasonic soft markers compared with karyotype analysis. There was a significant difference in detection rates of chromosomal abnormality between CMA and karyotype analysis in the single ultrasonic abnormality group, but none in the multiple ultrasonic abnormalities group.

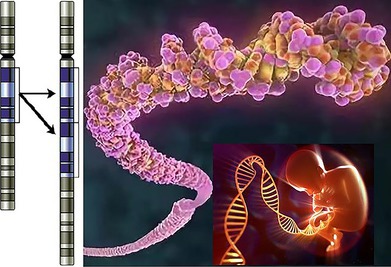

## 1 Introduction

Birth defects refer to external or internal abnormalities at birth, which can be divided into anatomical, functional, or metabolic abnormalities [1, 2]. The incidence of birth defects in China is about 5.6%, which is higher than that of some developed countries in the world [3]. There are some high‐risk factors for the occurrence of fetal birth defects, which are mainly caused by fetal genetic changes, followed by maternal factors, and also unknown causes [4, 5, 6]. At present, such technologies as regular prenatal examinations, prenatal ultrasound examinations, prenatal magnetic resonance imaging (MRI) examinations, maternal serology tests, and prenatal genetic examinations can effectively reduce the possibility of birth defects.

Fetal ultrasound is mainly used to evaluate fetal growth and gestational age, and on this basis to screen the fetus for visible malformations or abnormalities [7, 8, 9]. The abnormal soft markers of fetal ultrasound mainly includedincreased nuchal translucency (NT) thickness, ventriculomegaly, posterior fossa malformations, choroid plexus cyst, nasal bone hypoplasia, ventricular punctate echo, pyelic separation, echogenic bowel, long bone short, and single umbilical artery [10, 11]. Relevant studies have shown that fetuses with trisomy 21, 18, or 13 syndrome are often accompanied by increased NT thickness [12, 13], and choroid plexus cysts are common in fetuses with trisomy 18 syndrome [14]. Therefore, it is of great significance to determine whether genetic examination should be carried out when the fetus with an abnormal ultrasonic soft index is in order to reduce the rate of birth defects.

Invasive prenatal diagnosis involves obtaining fetal samples and testing genetic information to determine whether the fetus has chromosomal abnormalities, including villocentesis, amniocentesis, and umbilical cord blood puncture [15, 16]. Chromosomal karyotype analysis can detect chromosomal number abnormalities, balanced translocations, inversions, and chromosomal structure abnormalities with copy number variants (CNVs) greater than 5Mb [17, 18]. At present, chromosomal karyotype analysis is still the first‐line method and the gold standard of prenatal chromosome diagnosis, but it is unable to identify abnormalities in tiny fragments of chromosomes [19, 20]. The detection of chromosomal microduplication/microdeletion requires high‐resolution detection technology, such as chromosomal microarray analysis (CMA) technology [21, 22]. CMA technology can be used to analyze the CNVs of chromosomes through high‐throughput specific DNA probes, especially for the detection of microdeletion and microduplication [23].

In this study, a retrospective analysis was performed on fetuses with abnormal ultrasound soft markers that had undergone genetic testing. In this study, we analyzed the difference in the detection rate of chromosomal abnormalities in fetuses with abnormal ultrasound soft markers in chromosomal karyotype analysis and CMA, evaluated the significance of CMA for fetuses with abnormal ultrasound soft markers, clarified the relationship between abnormal ultrasound soft markers and chromosomal abnormalities, and provided valuable information for the pregnancy management of fetuses with abnormal ultrasound soft markers.

## 2 Materials and Methods

### 2.1 Study Cohort and Data Collection

A retrospective study was conducted on 193 fetuses with abnormal ultrasound soft markers who received interventional prenatal diagnosis in the Prenatal Diagnosis Center of Meizhou People's Hospital from October 2022 to February 2024. This study was approved by the Medical Ethics Committee of Meizhou People's Hospital. Genetic detection of fetal specimens (villi, amniotic fluid and umbilical cord blood) obtained by ultrasound‐guided puncture was carried out using G‐banding karyotype analysis and CMA (Affymetrix Cytoscan 750 K). According to the American College of Medical Genetics and Genomics (ACMG) guidelines, the clinical significance of CNVs is divided into five grades: pathogenic (P) CNV, likely pathogenic (LP) CNV, variants of uncertain significance (VUS) CNV, likely benign (LB) CNV, and benign (B) CNV [24, 25].

In this study, abnormal ultrasound soft markers include increased NT thickness, ventriculomegaly, nasal bone hypoplasia, choroid plexus cyst, pyelic separation, echogenic bowel, single umbilical artery, persistent left superior vena cava, and persistent right umbilical vein.

The diagnostic criteria of abnormal ultrasound soft markers [11, 26]:

1. *Increased NT thickening*: 11–13^+6^ weeks of pregnancy, NT ≥ 3.0 mm;
2. *Ventriculomegaly*: the width of the front or back foot of the lateral ventricle at any gestational week > 10 mm;
3. *Nasal bone hypoplasia*: no ossified nasal bone can be detected, or the nasal bone is short in length;
4. *Choroid plexus cyst*: the strong echo in the lateral ventricle at 16–24 weeks, and the echoless cystic structure in the choroid plexus;
5. *Pyelic separation*: the anterior and posterior pelvic diameters were separated between 0.6–1.0 cm;
6. *Echogenic bowel*: a bowel echo equal to or greater than the surrounding fetal skeletal echo.
7. *Single umbilical artery*: only one artery is seen on both sides of the bladder;
8. *Persistent left superior vena cava*: dysplasia of the communicating branches of the anterior main veins on both sides, the development of the Cuvier canal, forming a persistent left superior vena cava.
9. *Persistent right umbilical vein*: the right umbilical vein persisted, while the left umbilical vein was occluded.

### 2.2 | Statistical Analysis

SPSS 26.0 software was used for data analysis. Comparison between groups was tested by Chi‐square test or Fisher's exact test. *p* < 0.05 was set as statistically significant.

## 3 Results

### 3.1 Age of Pregnant Women and General Characteristics of the Fetuses

Of the 193 fetuses included in the study, there were 162 (83.9%) fetuses whose mothers were aged < 35 years old, and 31 (16.1%) fetuses whose mothers were ≥ 35 years old. there were 50 (25.9%), 132 (68.4%), and 11 (5.7%) fetuses with gestational weeks ≤ 13 weeks, 14–28 weeks, and > 28 weeks, respectively, according to the gestational week at the time of discovery of fetal abnormalities. In this study, fetal samples for genetic testing included 46 (23.8%) villus, 146 (75.6%) amniotic fluid, and 1 (0.5%) cord blood (Table 1).

**TABLE 1:** Age of pregnant women and general characteristics of the fetuses.

Characteristics	All cases (*n* = 193)
Age of mothers who had abortions (years)	
< 35, *n* (%)	162 (83.9%)
≥ 35, *n* (%)	31 (16.1%)
Gestational week at the time of discovery of fetal abnormalities (weeks)	
≤ 13, *n* (%)	50 (25.9%)
14–28, *n* (%)	132 (68.4%)
> 28, *n* (%)	11 (5.7%)
Type of samples tested by invasive prenatal diagnosis	
Villus, *n* (%)	47 (24.4%)
Amniotic fluid, *n* (%)	145 (75.1%)
Cord blood, *n* (%)	1 (0.5%)

### 3.2 The Relationship Between Abnormalities of Fetal Ultrasound Soft Markers and Chromosomal Abnormalities

In this study, in the fetuses with abnormal ultrasound soft markers, there were 77 (39.9%) fetuses with increased NT thickness, 33 (17.1%) fetuses with ventriculomegaly, 29 (15.0%) fetuses with nasal bone hypoplasia, 11 (5.7%) fetuses with choroid plexus cyst, 11 (5.7%) fetuses with pyelic separation, 6 (3.1%) fetuses with echogenic bowel, 5 (2.6%) fetuses with single umbilical artery, 4 (2.1%) fetuses with persistent left superior vena cava, and 3 (1.6%) fetuses with persistent right umbilical vein. The rest were fetuses with multiple abnormalities in ultrasound soft markers (Table 2).

**TABLE 2:** The relationship between abnormalities of fetal ultrasound soft markers and chromosomal abnormalities.

Ultrasound soft markers abnormalities	*n* (%)^a^	CMA				Karyotype		
		Aneuploidy	P/LP CNVs	VUS	Detection rate	Aneuploidy	Abnormal chromosomal karyotype	Detection rate
Increased NT thickness	77 (39.9%)	3	5	10	10.4%	3	0	3.9%
Ventriculomegaly	33 (17.1%)	0	3	5	9.1%	0	0	0
Nasal bone hypoplasia	29 (15.0%)	3	1	2	13.8%	3	0	10.3%
Choroid plexus cyst	11 (5.7%)	0	0	0	0	0	1	9.1%
Pyelic separation	11 (5.7%)	0	1	2	9.1%	0	0	0
Echogenic bowel	6 (3.1%)	0	0	1	0	0	0	0
Single umbilical artery	5 (2.6%)	0	0	0	0	0	0	0
Persistent left superior vena cava	4 (2.1%)	0	0	0	0	0	0	0
Persistent right umbilical vein	3 (1.6%)	0	0	0	0	0	0	0
Increased NT thickening + Nasal bone hypoplasia	6 (3.1%)	4	0	0	66.7%	4	0	66.7%
Increased NT thickening + Choroid plexus cyst	2 (1.0%)	0	0	0	0	0	0	0
Increased NT thickening + Single umbilical artery	1 (0.5%)	0	1	0	100.0%	0	0	0
Ventriculomegaly + Pyelic separation	1 (0.5%)	0	0	0	0	0	0	0
Nasal bone hypoplasia + Choroid plexus cyst	1 (0.5%)	0	0	0	0	0	0	0
Nasal bone hypoplasia + Single umbilical artery	1 (0.5%)	0	0	0	0	0	0	0
Nasal bone hypoplasia + Echogenic bowel	1 (0.5%)	0	0	0	0	0	0	0
Choroid plexus cyst + Pyelic separation	1 (0.5%)	0	0	0	0	0	0	0
Total	193 (100.0%)	10	11	20	10.9%	10	1	5.7%

Abbreviations: CMA, chromosome microarray analysis; CNV, copy number variant; P/LP CNV, Pathogenic/Likely pathogenic CNV; VUS, variants of uncertain significance.

^a^ Constituent ratio.

Chromosome aneuploidy was mainly found in fetuses with increased NT thickness or/and nasal bone hypoplasia, while P/LP CNVs were mainly concentrated in fetuses with increased NT thickness or ventriculomegaly. The detection rate of chromosomal karyotype was 5.7% (11/193), the detection rate of aneuploidy plus P/LP CNVs in fetuses with abnormal ultrasonic soft markers by CMA was 10.9% (21/193), and the additional diagnosis rate of CMA was 5.2%. Those with only 1 case (not representative) of the types of abnormal ultrasonic soft markers were excluded. After removing these cases, CMA had the highest diagnosis rate in fetuses with increased NT thickness and nasal bone hypoplasia simultaneously (66.7%), followed by nasal bone hypoplasia (13.8%), increased NT thickness (10.4%), ventriculomegaly (9.1%), and pyelic separation (9.1%). And chromosomal karyotype had the highest diagnosis rate in fetuses with increased NT thickness and nasal bone hypoplasia simultaneously (66.7%), followed by nasal bone hypoplasia (10.3%), choroid plexus cyst (9.1%), and increased NT thickness (3.9%) (Table 2).

### 3.3 Comparison of the Detection Rates of CMA and Karyotype Analysis Among Different Types of Abnormal Fetal Ultrasound Soft Markers and Different Numbers of Abnormal Ultrasonic Items

The difference between the detection rate of CMA and karyotype analysis was not statistically significant between different single ultrasound soft marker abnormalities. In the single ultrasonic abnormality group, the chromosomal abnormality detection rate of CMA was higher than that of karyotype analysis (8.9% vs. 3.9%, *p* = 0.041). However, the difference was not statistically significant in the multiple ultrasonic abnormalities group (*p* = 0.686) (Table 3).

**TABLE 3:** Comparison of the detection rates of CMA and karyotype analysis among different types of abnormal fetal ultrasound soft markers and different numbers of abnormal ultrasonic items.

Groups	Number of cases	Aneuploidy and P/LP CNVs		
		CMA	Karyotype	*p*
Types of abnormal ultrasound soft markers				
Increased NT thickness	77	8 (10.4%)	3 (3.9%)	0.118
Ventriculomegaly	33	3 (9.1%)	0 (0)	0.076
Nasal bone hypoplasia	29	4 (13.8%)	3 (10.3%)	0.687
Choroid plexus cyst	11	0	1 (9.1%)	0.306
Pyelic separation	11	1 (9.1%)	0	0.306
Echogenic bowel	6	0	0	—
Single umbilical artery	5	0	0	—
Persistent left superior vena cava	4	0	0	—
Persistent right umbilical vein	3	0	0	—
Number of abnormal ultrasonic items				
Single abnormality	179	16 (8.9%)	7 (3.9%)	0.041
Multiple abnormalities	14	5 (35.7%)	4 (28.6%)	0.686

Abbreviations: CMA, chromosome microarray analysis; CNV, copy number variant; P/LP CNV, Pathogenic/Likely pathogenic CNV.

### 3.4 Comparison of Chromosomal Abnormality Rate Among Different Abnormal Fetal Ultrasound Types and Groups of Different Numbers of Abnormal Ultrasonic Items

In this study, there were 11 cases with LP/P CNVs detected by CMA, but a normal chromosomal karyotype. There were 6 cases with increased NT thickness, 3 cases with ventriculomegaly, 1 case with nasal bone hypoplasia, and 1 case with pyelic separation. In these cases, eight of the cases had deletion CNVs with fragment sizes ranging from 112Kb to 14.20 Mb, and three of the cases had duplication CNVs with fragment sizes ranging from 990Kb to 46.28 Mb. In addition, 1 fetus with a choroid plexus cyst had mos 45,X [20]/47,XXX [13]/46,XX [17] detected by karyotype analysis, but the result of CMA was normal. It suggests that the detection of mosaicism by CMA is influenced by the mosaicism ratio (Table 4).

**TABLE 4:** The clinical data of 22 fetuses with aneuploidy and P/LP CNVs were detected by karyotype and/or CMA analysis.

Number	Age of pregnant woman (years)	Gestational week at the time of discovery of fetal abnormalities (weeks)	Type of samples tested by invasive prenatal diagnosis	Ultrasound of the fetuses	Karyotype	CMA	Clinical significance of CNVs
1	33	12^+3^	Villus	Increased NT thickness	47,XN,+21	arr21q11.2‐q22.3 (15016487–48,093,361) × 3 (33.08 Mb)	Pathogenic
2	28	12	Villus	Increased NT thickness	47,XN,+21	arr21q11.2‐q22.3 (15016487–48,093,361) × 3 (33.08 Mb)	Pathogenic
3	32	26	Amniotic fluid	Increased NT thickness	47,XN,+21	arr21q11.2‐q22.3 (15016487–48,093,361) × 3 (33.08 Mb)	Pathogenic
4	31	12^+3^	Villus	Increased NT thickness	47,XN,+18	arr18p11.32‐q23 (136228–78,013,728) × 3 (77.88 Mb)	Pathogenic
5	22	13	Villus	Increased NT thickness	47,XN,+18	arr18p11.32‐q23 (136228–78,013,728) × 3 (77.88 Mb)	Pathogenic
6	33	12^+4^	Villus	Increased NT thickness	mos 47,XN,+9[80]/46,XN [20]	arr9p24.3‐q34.3 (208455–141,018,648) × 2 ~ 3 (140.81 Mb mos)	Pathogenic
7	29	12^+2^	Villus	Increased NT thickness	46,XN	arr16p13.11 (15338153_16327887) × 3 (990Kb)	Likely pathogenic
8	26	12^+6^	Villus	Increased NT thickness	46,XN	arr16p11.2 (29428532–30,190,029) × 1 (761Kb)	Pathogenic
9	27	12	Villus	Increased NT thickness	46,XN	arr5p15.33p11 (113577–46,389,261) × 3 (46.28 Mb)	Pathogenic
10	35	23^+3^	Amniotic fluid	Increased NT thickness	46,XN	arr15q11.2 (22770422–23,288,350) × 1 (518Kb)	Pathogenic
11	31	12^+2^	Amniotic fluid	Increased NT thickness	47,XN,+21	arr21q11.2‐q22.3 (15016487–48,093,361) × 3 (33.08 Mb)	Pathogenic
13	29	18^+1^	Amniotic fluid	Increased NT thickness	46,XN	arrXp22.31 (6455152–8,135,568) × 1 (1.68 Mb)	Pathogenic
14	33	12	Villus	Increased NT thickness	46,XN	arr10q26.11q26.3 (121223476–135,426,386) × 1 (14.20 Mb)	Pathogenic
15	32	21^+1^	Amniotic fluid	Nasal bone hypoplasia	47,X,inv.(Y)(p11.2q11.2),+21	arr21q11.2‐q22.3 (15016487–48,093,361) × 3 (33.08 Mb)	Pathogenic
16	37	27^+5^	Amniotic fluid	Nasal bone hypoplasia	47,XN,+18	arr18p11.32‐q23 (136228–78,013,728) × 3 (77.88 Mb)	Pathogenic
17	27	19^+6^	Amniotic fluid	Nasal bone hypoplasia	46,XN	arr15q11.2 (22770422–23,082,237) × 1 (312Kb)	Pathogenic
18	36	16	Amniotic fluid	Nasal bone hypoplasia	47,XN,+21	arr21q11.2‐q22.3 (15016487–48,093,361) × 3 (33.08 Mb)	Pathogenic
19	30	27	Amniotic fluid	Ventriculomegaly	46,XN	arr7q11.23 (72682113–74,030,629) × 3 (1.35 Mb)	Pathogenic
20	29	16^+5^	Amniotic fluid	Ventriculomegaly	46,XN	arr15q24.2 (75601120_75768740) × 1 (168Kb)	Pathogenic
21	23	28	Amniotic fluid	Ventriculomegaly	46,XN	arrXp22.31 (6449837–8,143,509) × 1 (1.69 Mb)	Pathogenic
22	30	16	Villus	Choroid plexus cyst	mos 45,X [20]/47,XXX [13]/46,XX [17]	arr (1–22) × 2, (X,N) × 1	
23	27	21^+5^	Amniotic fluid	Pyelic separation	46,XN	arrXp21.1 (31713418–31,825,390) × 0 (112Kb)	Pathogenic

Abbreviations: CMA, chromosome microarray analysis; CNV, copy number variant; P/LP CNV, Pathogenic/Likely pathogenic CNV.

## 4 Discussion

Ultrasound soft index refers to the small non‐specific variation of fetal structure found in prenatal ultrasound examination. Although most of the soft markers abnormalities are transient manifestations during pregnancy, they are also risk indicators indicating chromosomal abnormalities [27, 28]. For example, the proportion of increased NT thickness and choroid plexus cysts in some children with trisomy syndrome was significantly higher than that in the control population [12, 13, 14]. Importantly, different indicators of ultrasound abnormalities may be associated with different chromosomal abnormalities. For example, different types of fetal growth restriction were associated with different types of chromosomal number abnormalities [29]. Hu et al. suggested that the potential chromosomal aberrations of different types of ultrasound soft markers vary greatly [26]. Therefore, genetic factors play an important role in the occurrence of ultrasound soft index abnormalities, and exploring the genetic causes through cytogenetics and molecular genetics technology provides an important guarantee for the prevention of birth defects.

Karyotyping is still the standard method for prenatal cytogenetic diagnosis and can detect aneuploidy, deletion/duplication of large fragments, and chromosomal structural recombination. However, its resolution is low and it can only detect changes in genetic material with fragment sizes above 5 to 10Mb [30]. At present, many studies have explained the relationship between ultrasonic soft index abnormality and chromosomal abnormality through cell karyotype analysis. Studies have suggested that ultrasound soft markers have a certain correlation with fetal aneuploidy or genetic syndrome, and can be used to indicate the risk of trisomy 21, trisomy 18, trisomy 13, and 45, XO [31, 32]. Abele et al. reported that increased NT thickness was significantly related to chromosomal abnormalities, and the proportion of increased NT thickness in fetuses with trisomy 21 reached 71% [33]. According to the studies of McKechnie et al., the detection rate of abnormal karyotype in fetuses with ventriculomegaly was 3%–10% [34]. Wegrzyn et al. found that the rate of nasal bone hypoplasia in fetuses with trisomy 21 was 64.8%, which was significantly higher than 4.3% of fetuses with a normal karyotype [35]. Other studies have also shown that the incidence of chromosomal abnormalities in fetuses with nasal bone hypoplasia is 21.7%–51.7%, of which trisomy 21 is the most common [36, 37]. Granese et al. studied 61 fetuses with a single umbilical artery and found that the incidence of chromosomal abnormality in a single umbilical artery is 2.56%, and when a single umbilical artery is combined with other abnormalities, the incidence of chromosomal abnormality is as high as 41.6% [38]. Dagklis et al. showed that in the karyotype analysis of a simple single umbilical artery, the incidence of chromosomal abnormality was 4.2% [39]. However, some literature pointed out that choroid plexus cyst, pyelectasis, echogenic bowel, and other markers were often transient and not necessarily related to chromosomal abnormalities [40, 41]. It can be seen that chromosomal abnormalities are considered to be one of the causes of abnormalities of ultrasound soft markers, but other chromosomal structural abnormalities, especially those that cannot be detected by karyotype analysis, should not be ignored.

CMA is a high‐resolution, high‐throughput molecular karyotype analysis technique for the detection of human genome CNVs. Chromosomal aneuploidy was common in fetuses with increased NT thickness, and the incidence of pathogenic CNVs was significantly higher in fetuses with multiple soft marker abnormalities and short femur length [26, 42]. Akalın et al. reported that P/LP CNVs were detected in 6.5% of fetuses with ultrasound soft markers [43]. The detection rate of abnormal chromosome in fetuses with abnormal ultrasound soft marker/markers was 5.0% in the study of Jiang et al. [44].

Compared with traditional karyotyping, CMA can detect more clinically significant genetic abnormalities [45, 46]. CMA can improve the diagnostic rate of fetal chromosomal abnormalities with abnormal ultrasound soft markers [47]. Lu et al. found that CMA additionally detected 5 pathogenic CNVs in 160 fetuses with abnormal soft markers and normal karyotype [48]. Pan et al. performed CMA detection on 122 fetuses of NT > 3.5 mm with normal karyotype, and 7 (5.7%) pathogenic CNVs were detected [49]. Leung et al. found that the incidence of microdeletion/microduplication in increased NT thickened fetuses with normal karyotype was about 10%, and the incidence of microdeletion/microduplication was higher when other ultrasonic structural abnormalities were combined [50]. Bornstein et al. reported that the overall detection rate of pathogenic CNVs was 4.3% in fetuses with increased NT thickness, 1.6% in cases with simple increased NT thickness, and 5.9% when combined with other abnormalities [51]. Dukhovny et al. tested 57 fetuses with nasal bone hypoplasia, and 3 (5.3%) microdeletions/microduplications were detected by CMA in fetuses with normal karyotype [52].

Therefore, CMA can detect more pathogenic variants than chromosomal karyotype analysis [53]. However, the limitations of CMA have to be mentioned; CMA cannot detect a low proportion of chromosomal mosaicism and chromosomal balanced translocations [54]. It is suggested that chromosomal karyotype plus CMA should be performed on fetuses with abnormal ultrasound soft markers. The two methods complement each other to improve the detection rate and accuracy of abnormal results. In addition, the occurrence and development of ultrasound soft markers abnormalities is a very complicated process in which genetic factors play an important role. It is believed that with the application of more advanced and lower cost sequencing technology in prenatal diagnosis, more genetic factors will be detected at the gene level.

Chromosomal karyotype analysis and CMA detection are routine items in the genetic detection of fetuses with ultrasound abnormalities. Chromosomal abnormalities were detected in about 32% of fetuses with abnormal ultrasound, and CMA had an additional detection rate of 4%–6% in fetuses with a normal chromosomal karyotype [55]. Therefore, the genetic cause of more than 60% of fetuses with abnormal ultrasound remains unknown. In recent years, high‐throughput sequencing technology has been gradually applied to the detection of congenital diseases, which has greatly improved the diagnostic expectations of genetic diseases, and the research and guidance on the application of whole exome sequencing (WES) technology in prenatal diagnosis have been continuously formed and accumulated [56]. Studies suggest that WES can increase the detection rate by 8.5%–10% in fetuses with no abnormality in chromosomal karyotype and CMA analysis [57]. In this study, pregnant women's compliance with WES testing was poor, the number of fetuses undergoing WES testing was small, and the obtained test data was insufficient for statistical analysis. Therefore, the application value of WES in prenatal diagnosis still needs more research to reveal.

In this study, the genetic test results of 193 fetuses with abnormal ultrasound soft markers were analyzed, and the results showed that CMA had a higher detection rate than chromosomal karyotype analysis. However, there are still shortcomings in this study. The number of fetuses with abnormal ultrasound soft markers included is small, such as echogenic bowel, single umbilical artery, persistent left superior vena cava, and persistent right umbilical vein. Secondly, we failed to collect data on other abnormal ultrasound soft markers, such as widening of posterior fossa cisterna and short long bone. Therefore, more case data will need to be collected in the future to further analyze the relevant results.

## 5 Conclusions

CMA can significantly improve the detection rate of chromosomal abnormalities in fetuses with abnormal ultrasonic soft markers compared with karyotype analysis. There was a significant difference in detection rates of chromosomal abnormality between CMA and karyotype analysis in the single ultrasonic abnormality group, but none in the multiple ultrasonic abnormalities group.

## Author Contributions

Lina Liu and Heming Wu designed the study. Lina Liu, Lingna She, Zhiyuan Zheng, Shuxian Huang, and Heming Wu collected clinical data. Lina Liu and Heming Wu analyzed the data. Lina Liu and Heming Wu prepared the manuscript. All authors were responsible for critical revisions, and all authors read and approved the final version of this work.

## Conflicts of Interest

The authors declare no conflicts of interest.

## Data Availability

The data that support the findings of this study are available on request from the corresponding author.
